# Resolution of Severe Neurologic Signs Following Intravenous Lipid Emulsion Therapy in a Young Dog With a Portosystemic Shunt: Case Report

**DOI:** 10.3389/fvets.2021.798198

**Published:** 2021-12-08

**Authors:** Amanda M. Spillane, Jenica L. Haraschak, Maureen A. McMichael

**Affiliations:** ^1^Department of Veterinary Clinical Medicine, University of Illinois at Urbana-Champaign, Urbana, IL, United States; ^2^Department of Clinical Sciences, College of Veterinary Medicine, Auburn University, Auburn, AL, United States

**Keywords:** intralipid, intravenous fat emulsion, hepatic encephalopathy, ammonia, case report

## Abstract

A 5-month-old male intact Great Pyrenees was presented for an acute onset of severe neurologic signs (stupor, absent menace, intermittent head turn to the left). The patient's history included possible naproxen ingestion with a maximum ingested dose of 59 mg/kg, exceeding the reported dose of >50 mg/kg known to cause neurologic signs. Blood sampling for baseline bloodwork was performed, and intravenous lipid emulsion (ILE) was subsequently administered, for treatment of the suspected toxicosis. Due to severe and life-threatening neurologic signs, other methods of decontamination were contraindicated and unlikely to be effective; extracorporeal therapy was also unavailable. Complete resolution of neurologic signs occurred 30 min after completion of ILE therapy. At this time, the owners found the missing naproxen tablets after returning home and the bloodwork results returned revealing findings consistent with hepatic encephalopathy. The fasted blood ammonia concentration immediately prior to ILE administration was 702.1 μg/dL (reference interval, RI: 24–36 μg/dL) and decreased to 194.1 μg/dL 24 h later. In the first 24 h, the patient also received three doses of lactulose, N-acetylcysteine, and intravenous fluids. The patient was subsequently diagnosed with a single, large intrahepatic portosystemic shunt *via* computed tomography and underwent an endovascular coil embolization procedure. Given the rapid and dramatic improvement in severe neurologic signs after ILE therapy alone, it is strongly suspected that this treatment resulted in improvement of hepatic encephalopathy.

## Introduction

Hepatic encephalopathy (HE) is a spectrum of neurologic abnormalities seen in patients with moderate to severe liver dysfunction (1, 2). Severity can range from subtle and episodic to severe clinical signs including altered mentation (obtunded, stuporous, comatose), ataxia, blindness, muscle tremors, and focal or generalized seizures (2, 3). The exact mechanism of HE is not fully understood, with suggested mechanisms including an altered blood-brain-barrier (4–6), cerebral blood flow or cerebral energy metabolism, changes in central and neuromuscular neurotransmission, amino acid imbalance, or oxidative and neuroinflammatory damage (3, 4, 6–8). It is widely accepted that hyperammonemia is a key component in the development of HE (3, 5, 6, 8). Ammonia is highly lipophilic and easily crosses cell membranes, including the blood-brain barrier (5, 7, 8). Ammonia is produced as the end product of protein metabolism in the intestinal tract (2, 4, 6). Ammonia is absorbed from the intestinal tract into the portal circulation, where it is converted to ammonium (NH${}_{4}^{+}$) and transported to the liver for rapid detoxification (3, 4, 6). Blood ammonia concentration increases with hepatic dysfunction or when the portal circulation bypasses the liver, and is the only readily measurable analyte implicated in the pathogenesis of HE (9–12).

A portosystemic shunt (PSS) is a vascular anomaly that allows portal blood to bypass the hepatic parenchyma and directly enter the systemic circulation (13–15). As blood bypasses the hepatic parenchyma, toxic substances such as ammonia, mercaptans, free fatty acids, phenols, and bile salts accumulate within the systemic circulation and can lead to HE (2). If possible, attenuation of the shunting vessel is the treatment of choice for PSS, as hepatic function will continue to decline until blood flow is redirected back to the liver (13). Medical management is necessary when complete shunt closure is not achieved, to stabilize signs prior to surgical attenuation, or when surgical attenuation is either not feasible or contraindicated. Medical management aims to reduce intestinal ammonia production and absorption, involving dietary modification, cathartic administration, and antibiotic therapy (2, 13, 16).

Intravenous lipid emulsion (ILE) has been used for treatment of various lipophilic drug toxicoses including local anesthetics, macrocyclic lactones, psychotropic drugs, organophosphates, permethrin, baclofen, bromethalin, naproxen, ibuprofen, and phenobarbital (17, 18). As ammonia is a lipophilic compound, it is possible that ILE administration could lower brain ammonia concentration, by drawing ammonia into the intravascular space to bind with ILE and promote its excretion. In this case report, ILE was administered due to an initial suspicion of naproxen toxicosis in a dog that was later confirmed to have severe HE. Naproxen exposure was definitively eliminated as a possibility when the owners found the missing tablets after returning home the same day, however ILE administration resulted in an unexpected, positive outcome in this patient. To the authors' knowledge, ILE has never been implicated for use in HE, in either human or veterinary medicine. The purpose of this case report is to describe the improvement of severe neurologic signs in a young dog with a portosystemic shunt, following administration of ILE therapy.

## Patient Information

A 5-month-old male intact Great Pyrenees weighing 17 kg presented to a tertiary referral hospital for an acute onset of progressive neurologic signs, anorexia, and vomiting. The patient had a history of intermittent vomiting for 1 month. Twelve hours prior to presentation he became anorexic, was hypersalivating and pacing with a slightly uncoordinated gait. The morning of presentation the patient was dull and severely ataxic, and progressively became non-ambulatory and stuporous. The history included possible ingestion of multiple naproxen tablets with a maximum ingested dose of ~59 mg/kg. After the dog was admitted and received ILE therapy, the owners returned home and found the missing naproxen tablets, making naproxen toxicosis unlikely.

## Clinical Findings

On initial examination, the dog was stuporous and had severe ptyalism. Vital parameters were noted to be mildly elevated (heart rate 120 beats/min, respiratory rate 48 breaths/min, rectal temperature was 39.8°C [103.6°F], Doppler blood pressure 170 mmHg). On neurologic examination, the dog was stuporous, non-ambulatory, exhibited intermittent head turn to the left, and was non-visual determined by negative cotton-ball tracking and bilaterally absent menace response.

## Timeline

**Figure 1:**
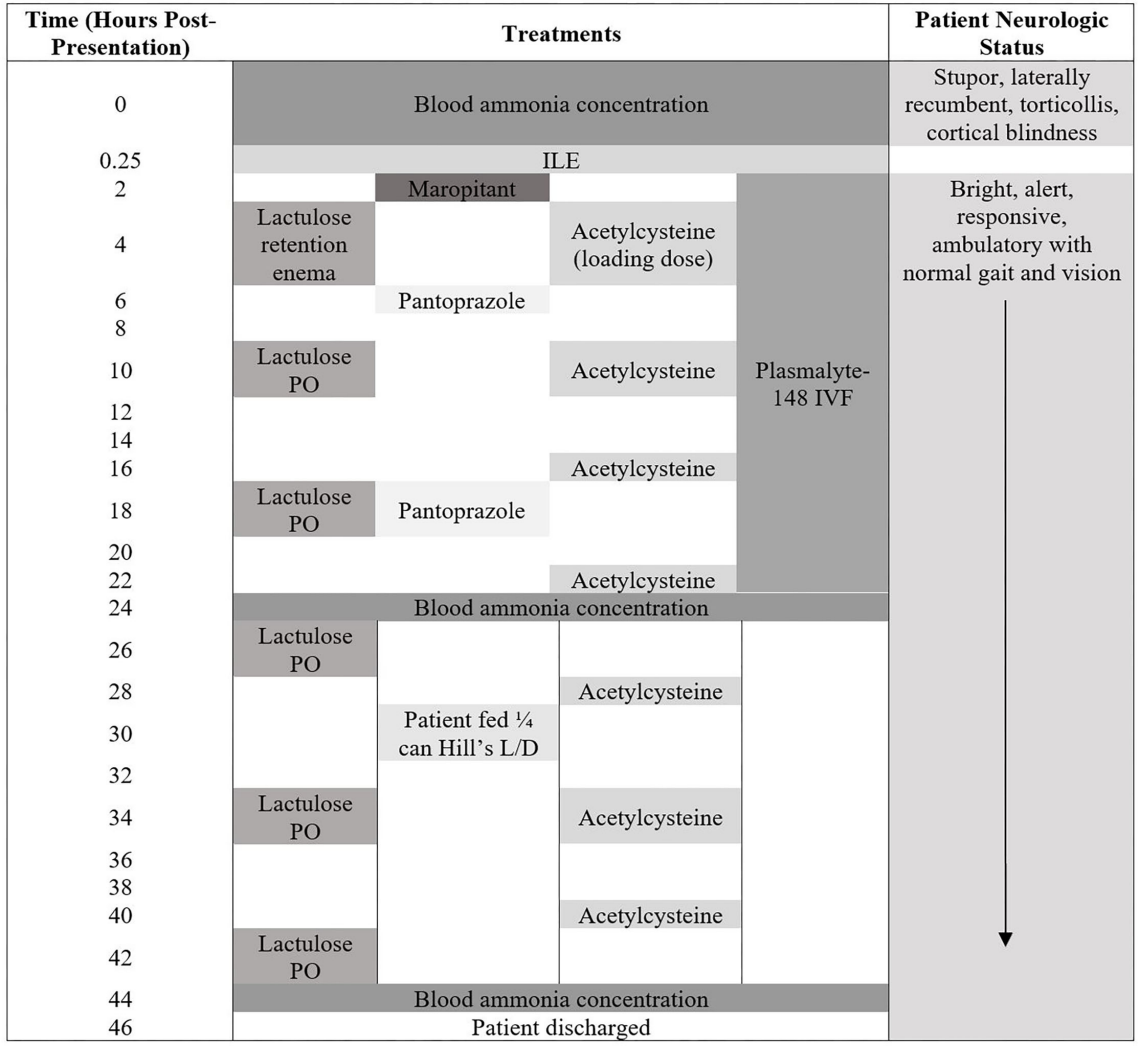
Patient neurologic status and treatments administered from the time of presentation until hospital discharge.

The timeline of the treatments administered, and patient neurologic status is presented in Figure 1.

## Diagnostic Assessment

**Figure 2:**
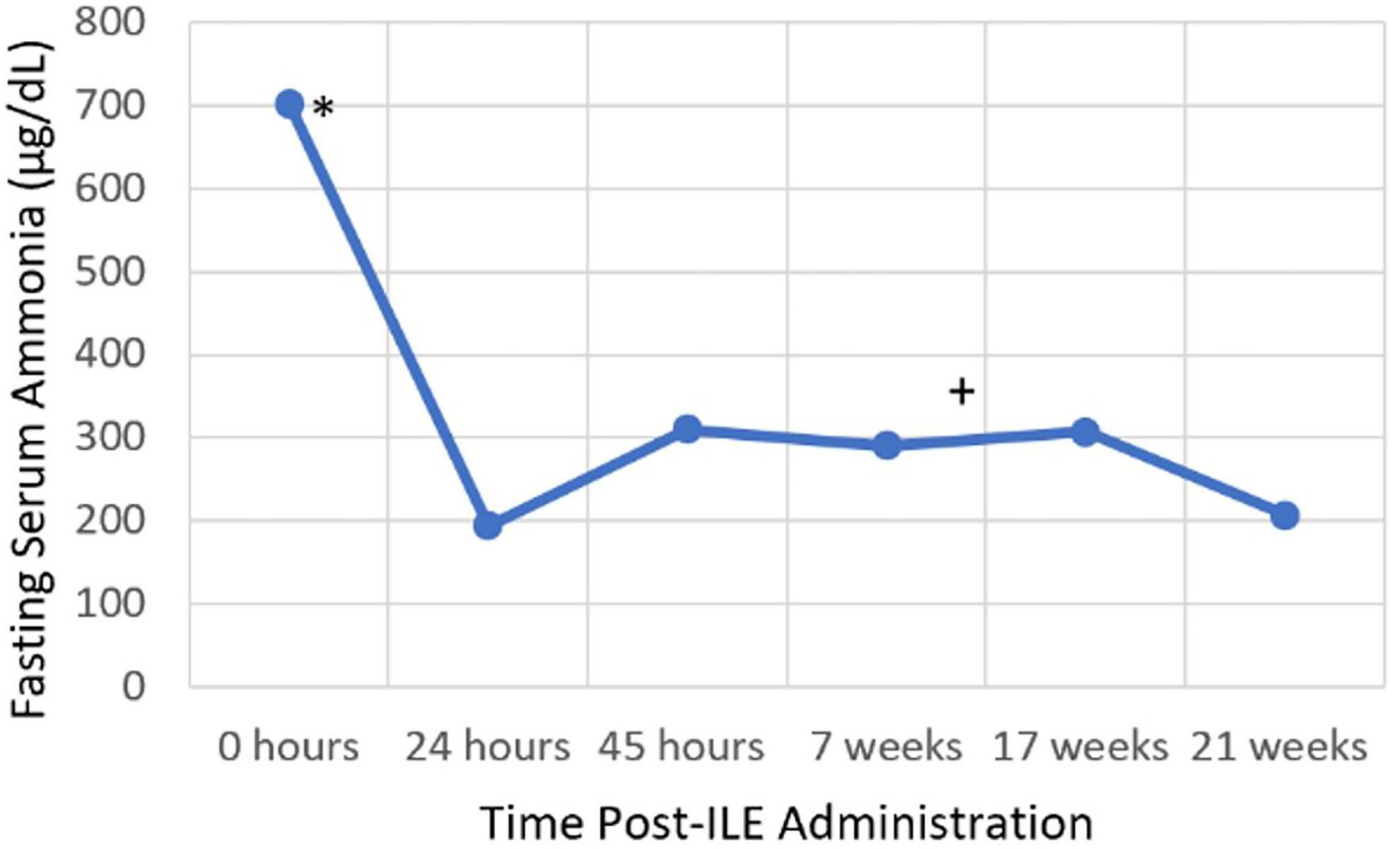
Serial blood ammonia concentration. *Intravenous lipid emulsion administration, ^+^Endovascular coil embolization.

Venous blood gas findings on presentation are presented in Table 1. Blood sampling for CBC, chemistry, blood ammonia concentration, prothrombin time (PT), and partial thromboplastin time (PTT) was performed, prior to administration of any treatment. CBC and chemistry abnormalities are presented in Table 2. The patient was markedly hyperammonemic [702.1 μg/dL (RI: 24–36 μg/dL); Beckman Coulter Analyzer AU680] (Figure 2). The patient had been fasted for 17 h prior to measuring the blood ammonia concentration. The results of this bloodwork were returned after the completion of ILE. The PT and PTT both returned within the reference range [PT 9.5 s (RI: 6.0–10.0 s), PTT 14.6 s (RI: 9.0–15.0 s)].

**Table 1:** Venous blood gas findings on presentation to the emergency room.

**Parameter**	**Value**	**Reference interval**
HCT (%)	35	38.13–53.75
pH	7.45	7.39–7.49
PCO_2_ (mmHg)	26.4	23.11–37.41
Bicarbonate (mmol/L)	18.4	17.08–24.68
Base excess (mmol/L)	−3.8	−4 to 0
Potassium (mmol/L)	4.83	3.7–4.91
Sodium (mmol/L)	151.4	144–151
Chloride (mmol/L)	120.8	110.04–117.96
BUN (mg/dL)	9	9.1–24.5
Creatinine (mg/dL)	0.6	0.73–1.19

**Table 2:** CBC and chemistry abnormalities on presentation to the emergency room.

**Parameter**	**Value**	**Reference interval**
HCT (%)	29.9	37.3–61.7
BUN (mg/dL)	5	7–29
Albumin (g/dL)	2.3	2.5–3.8
Alanine aminotransferase (U/L)	435	8–75
Alkaline phosphatase (U/L)	256	7–92
Sodium (mmol/L)	166	145–157
Chloride (mmol/L)	124	105–119

## Therapeutic Intervention

Based on the acute onset neurologic signs, and presumed exposure to naproxen at a dose of 59 mg/kg, naproxen toxicosis was initially suspected, as central nervous system signs have been reported at doses >50 mg/kg (19). ILE (Intralipid 20% I.V. Fat Emulsion; Fresenius Kabi) was administered while awaiting the bloodwork results, due to the suspicion for naproxen toxicosis and severe and life-threatening neurologic signs, making other routes of decontamination contraindicated and unlikely to be effective. A 1.5 mL/kg slow IV bolus of ILE was administered, followed by a constant rate infusion at 0.25 mL/kg/min IV for 45 min.

The patient was then admitted into the intensive care unit. At this time, the owners called to advise that they had located the missing naproxen tablets and that exposure was no longer a possibility. The following treatments were administered, listed in chronological order (Figure 1): maropitant (Cerenia Injectable Solution 10 mg/mL; Zoetis) at 1 mg/kg, IV, q 24 h, Plasmalyte-148 (Plasma-Lyte A 1,000 mL; Baxter Healthcare Corporation) at 60 mL/kg/day, N-Acetylcysteine (N-Acetylcysteine 200 mg/mL solution; Hospira Inc.) at 140 mg/kg, IV loading dose, once, then 70 mg/kg, IV, q 6 h, and pantoprazole (Pantoprazole 40 mg vial; West-Ward) at 1 mg/kg, IV, q 12 h. The patient received one lactulose (Lactulose solution 10 g/15 mL; Hi-Tech Pharmacal Co. Inc.) retention enema at 1.5 mL/kg diluted 1:3 with warm water following admission into ICU. Overnight the patient subsequently received two doses of lactulose at 0.25 mL/kg, PO, q 8 h.

## Follow-up and Outcomes

Within 30 min of completion of ILE, the patient showed complete resolution of all neurologic abnormalities. The dog became ambulatory with a normal gait and appeared to have normal vision, as he was able to navigate obstacles, track cotton-balls and had normal menace response bilaterally. His mentation improved to bright, alert, and responsive, with the patient tail wagging and intentionally barking for attention at passersby. The patient remained stable and neurologically normal thereafter.

The following day a blood ammonia concentration was repeated, ~24 h after initial blood ammonia concentration and was markedly decreased to 194.1 μg/dL (Figure 2). The patient was not fed between the first and second blood ammonia concentrations. Fasting and post-prandial bile acids were performed at this time and were both elevated: fasting bile acids 10.3 μmol/L (RI: 2.0–7.5 μmol/L), post-prandial bile acids 65.0 μmol/L (RI: 2.0–25.0 μmol/L). The patient was hospitalized for another 24 h and received the following treatments: Plasmalyte-148 at 60 mL/kg/day, N-Acetylcysteine at 70 mg/kg, IV, q 6 h, and lactulose at 0.25 mL/kg, PO, q 8 h. The patient was fed once in hospital (1/4 can wet Hill's L/D; Hill's Wet L/D Dog Food; Hill's Pet Nutrition Inc.), ~30 h following presentation, which was 14-h prior to the third ammonia concentration. The third blood ammonia concentration was repeated 44 h after initial presentation and returned as 310.3 μg/dL (Figure 2). The patient was discharged 46 h after initial presentation and was observed to be clinically normal. The patient was medically managed at home with a moderately protein restricted diet (Hill's L/D diet), lactulose at 0.25 mL/kg, PO, q 8 h, S-adenosyl-L-methionine (Denamarin 425 mg tablets; Nutramax Laboratories Veterinary Sciences Inc.) at 425 mg, PO, q 24 h, metronidazole (Metronidazole 50 mg/mL oral solution; authors' institution Veterinary Clinic Prescription Dispensary) at 10 mg/kg, PO, q 12 h and levetiracetam (Levetiracetam 500 mg tablets; Aurobindo Pharma Limited) at 30 mg/kg, PO, q 8 h.

Six weeks later, the patient was diagnosed with a large single, intrahepatic portosystemic shunt *via* computed tomography. Shunt attenuation was achieved *via* an endovascular coil embolization procedure 3 weeks later. After shunt attenuation was performed, the metronidazole was discontinued, but the patient continues to receive Hill's L/D diet, lactulose, levetiracetam, and S-adenosyl-L-methionine 2 years later. On two subsequent recheck exams, fasting blood ammonia concentrations remain elevated (206.5–307.5 μg/dL) (Figure 2), however with no observed clinical signs and the patient remains alive and clinically normal 2 years later.

## Discussion

This case report demonstrates an unexpected positive outcome, as ILE was administered for suspected naproxen toxicosis, however resulted in clinical resolution of severe neurologic signs in a dog with HE. No treatments apart from ILE were administered during the time between the patient being severely neurologically affected and clinically normal. Based on the severity of this patient's signs, it is unlikely that the patient experienced spontaneous resolution of signs without institution of treatment for HE. Given that ammonia is known to be lipophilic, and this patient experienced rapid and dramatic improvement in severe neurologic signs 30 min following completion of ILE, it is strongly suspected that that ILE was responsible for this clinical improvement, by drawing the ammonia out of the brain and into the lipid partition in the intravascular space. To the authors' knowledge, using ILE for the treatment of HE has never been reported in veterinary or human medical literature.

The dog was presented with severe, acute neurologic signs (stupor, head turn, blindness). The initial concern for the acute neurologic signs was naproxen toxicosis, given possible access to a dose of ~59 mg/kg. Naproxen is a propionic acid derivative non-steroidal anti-inflammatory drug used in human medicine for its anti-inflammatory, analgesic, and antipyretic properties. Clinical signs of canine naproxen toxicosis include gastrointestinal signs at doses >5 mg/kg, renal damage at doses >10–15 mg/kg and neurologic signs at doses >50 mg/kg, as reported by the ASPCA Animal Poison Control Center (APCC^1^). Due to the high possible ingested dose, narrow safety margin and severe clinical signs present, ILE administration was elected in this patient due to a prior case report demonstrating successful decontamination in naproxen overdose (19). Given the patient's severe neurologic signs, it was believed that gastrointestinal decontamination was contraindicated and that intravenous fluids and supportive care alone would have been inadequate given the severity of signs. Furthermore, there was no access to extracorporeal therapies at the institution, which has also been reported in the management of non-steroidal anti-inflammatory drug toxicoses (20–22). Following admission into ICU, naproxen exposure was eliminated as a possibility and the patient's laboratory findings returned, supporting a diagnosis of HE.

On presentation to the emergency department, the patient was exhibiting severe neurologic signs and was recorded as being markedly hyperammonemic. It is widely accepted that hyperammonemia plays a major role in the pathophysiology of hepatic encephalopathy, due to altered astrocyte function (3, 5, 6, 8). Due to the magnitude of the hyperammonemia, supporting bloodwork showing evidence of hepatic dysfunction, as well as neurologic signs localizing to the cerebrum and the improvement following treatment, it is presumed that hepatic encephalopathy was present. The marked clinical improvement following ILE administration was an unexpected finding for a patient with HE, as this has not been reported previously, to the authors' knowledge.

If ILE did successfully result in clinical resolution of severe neurologic signs in this patient with HE, then we suspect that this occurred through removal of ammonia from the brain, *via* the “lipid-sink” theory. This mechanism involves formation of a lipid partition in the blood plasma, which in turn causes movement of the lipid-soluble compound from the affected tissues into the intravascular space, where it binds with ILE to facilitate excretion (17, 18). ILE binds lipophilic substances with a log P >1.0 and has demonstrated success with many lipophilic compounds. While the log P of free ammonia *in vivo* is to the authors' knowledge unknown, it is widely accepted that ammonia is lipophilic (5, 7, 8). The ammonia level in the brain cannot be measured and directly correlated with a medication's ability to reduce cerebral ammonia levels, however the dramatic clinical improvement with no other therapy apart from ILE supports a beneficial effect. During the first 24 h of hospitalization, the patient's blood ammonia concentration also underwent dramatic reduction, which could be due to ILE therapy.

The patient's fasted blood ammonia level on presentation was markedly elevated and reduced by over 3.5-fold within 24 h. During this time, the patient only received one lactulose enema and two oral doses of lactulose. Lactulose, a type of cathartic, is a key component to medical management of hyperammonemia, and works by trapping ammonium ions in the gastrointestinal lumen and decreasing gastric transit time, thereby decreasing ammonia absorption (13). There are limited studies available that critically assess the efficacy of lactulose, however one observational study did not demonstrate a clinically proven benefit compared to dietary modification alone (16). During the time between the first and second measurements, it is possible that endogenous clearance of ammonia may have contributed to the reduction in blood ammonia concentration. The half-life of ammonia is only minutes in healthy subjects, however it is unknown in patients with hepatic dysfunction or portosystemic shunting (23). Based on the patient's clinical resolution with ILE therapy alone, limited lactulose treatment received and the other treatments having no reported effect on ammonia concentration, it is hypothesized that ILE administration may have contributed to the marked reduction in blood ammonia concentration. This could have occurred due to ammonia being bound to the lipid molecules and thereby not read by the ammonia analyzer, as it measures free ammonia. However, it is important to note that the patient's blood was not lipemic at the time of the 24-h blood ammonia concentration measurement. Alternatively, the lipid-bound ammonia may have already been excreted from the intravascular space, at the time the second blood ammonia concentration was performed. Furthermore, the second ammonia measurement after ILE administration was the lowest recorded ammonia level for this patient. Subsequent blood ammonia concentrations after prolonged medical management and endovascular coil embolization were higher than the post-ILE blood ammonia concentration. This may suggest that ILE was a successful method of short-term ammonia reduction, when used in combination with lactulose therapy. It is interesting to note that this patient remained persistently hyperammonemic after PSS coil embolization, which may be due to residual flow through the shunt vessel or continued hepatic dysfunction. It is also possible that ILE may have bound a toxin other than ammonia, resulting in clinical patient improvement but not complete resolution in the hyperammonemia. While other factors may have influenced the reduction in blood ammonia concentration, the dramatic improvement in neurologic status following ILE therapy alone is supportive that this treatment had a positive therapeutic impact.

If ILE is effective in reducing brain or blood ammonia levels, then its use in dogs with hyperammonemia may have multiple clinical benefits. It could provide potentially faster medical stabilization of signs related to HE. This would be desirable for patients with severe neurologic signs, that without prompt treatment may have increased risk for morbidity and mortality. A case report by Culler et al. reported on the use of therapeutic plasma exchange in a dog with severe HE, and how this therapy provided rapid improvement in clinical signs and reduction in hyperammonemia, allowing definitive shunt ligation to be performed sooner and in a patient with less severe signs of HE (24). ILE therapy may also provide a fast and effective means of acute reduction in signs of HE in severely affected patients, or those refractory to standard medical management, and is a more readily available and inexpensive treatment option, as extracorporeal therapies are not available in all hospitals. Stabilizing patients with severe HE prior to pursuing anesthesia for definitive diagnosis and treatment, is vital for improved patient outcome. It has been reported that 68% of dogs and 44% of cats undergoing surgical attenuation of a congenital PSS had preoperative neurological abnormalities (25, 26). Potential adverse effects of ILE are rare in veterinary medicine, but may include hypersensitivity reaction, bacterial contamination and infection, lipemia and subsequent pancreatitis, lipid embolization, and fat overload syndrome (17). The low risk of adverse effects, cost effectiveness, and potential benefits, could make ILE use in hyperammonemic dogs a promising future treatment, that should be investigated prospectively.

There are a few limitations to the case report. It is possible that sample collection or handling may have affected the ammonia concentration. All blood ammonia concentrations were run at the on-site, veterinary clinical pathology lab as soon as they were collected. Furthermore, the patient was fasted for at least 12 h prior to any of the blood ammonia concentrations reported in this paper. It is known that lipemia interferes with blood ammonia concentration measurement (27). It is recommended that blood samples are collected at least 5–6 h after the administration of Intralipid, to prevent lipemia interfering with test results (28). For this patient, blood samples were visually inspected for lipemia prior to blood ammonia concentration measurement. Despite these measures, it is still possible that sample collection or handling may have caused variability in the results, as ammonia is unstable *ex vivo* (5). Plasma ammonia was repeated 24 h after presentation, after the patient had also received lactulose and other medications, therefore making it difficult to attribute causation of ILE administration to lowered blood ammonia concentration. Given that this patient's severe neurologic signs completely resolved after ILE therapy alone, with no other treatments during this time, it is possible that ILE may have caused a reduction in blood ammonia level in conjunction with lactulose therapy.

In summary, ILE therapy alone resulted in a rapid and complete resolution of severe neurologic signs in a young dog with a portosystemic shunt. Additionally, the combination of lactulose and ILE resulted in a dramatic decrease in the ammonia level in this patient. As ILE is readily available, inexpensive, and has few reported adverse effects in veterinary medicine, it may be a promising future treatment option for treatment of hyperammonemia in conjunction with standard therapy for cases with acute, severe neurologic signs and should be investigated prospectively.

## Data Availability Statement

The original contributions presented in the study are included in the article/supplementary material, further inquiries can be directed to the corresponding author.

## Ethics Statement

Ethical review and approval was not required for the animal study as this was a retrospective case report. Written informed consent for participation was not obtained from the owners as this was a retrospective case report that did not require informed consent for research purposes.

## Author Contributions

AS: clinical management of the case, initial draft, critical revisions, and final manuscript approval. JH: clinical management of the case, critical revisions, and final manuscript approval. MM: clinical management of the case, critical revisions, and final manuscript approval. All authors contributed to the article and approved the submitted version.

## Conflict of Interest

The authors declare that the research was conducted in the absence of any commercial or financial relationships that could be construed as a potential conflict of interest.

## Publisher's Note

All claims expressed in this article are solely those of the authors and do not necessarily represent those of their affiliated organizations, or those of the publisher, the editors and the reviewers. Any product that may be evaluated in this article, or claim that may be made by its manufacturer, is not guaranteed or endorsed by the publisher.

## Footnotes

^1^APCC Animal Poison Control Center electronic medical record database.

## Abbreviations

HE: hepatic encephalopathy
ILE: intravenous lipid emulsion
IV: intravenous
PSS: portosystemic shunt
PT: prothrombin time
PTT: thromboplastin time
RI: reference interval.
